# On Shepard–Gupta-type operators

**DOI:** 10.1186/s13660-018-1823-7

**Published:** 2018-09-10

**Authors:** Umberto Amato, Biancamaria Della Vecchia

**Affiliations:** 1 Consiglio Nazionale delle Ricerche, Istituto per la Microelettronica e Microsistemi, Napoli, Italy; 2 Dipartimento di Matematica, Università degli Studi La Sapienza, Roma, Italy

**Keywords:** 41A36, 41A25, 94A08, Shepard operators, Gupta-type variant, Direct and converse results, Image compression

## Abstract

A Gupta-type variant of Shepard operators is introduced and convergence results and pointwise and uniform direct and converse approximation results are given. An application to image compression improving a previous algorithm is also discussed.

## Introduction

In the last decades Shepard operators have been object of several papers, thanks to their properties interesting in classical approximation theory and in scattered data interpolation problems. In particular Shepard operators are linear, positive, rational operators, of interpolatory-type, preserving constants and achieving approximation results not possible by polynomials. Pointwise and uniform approximation error estimates, converse results, bridge theorems, saturation statements, simultaneous approximation results can be found for example in [1–7]. Applications of Shepard operators to scattered data interpolation problems, image compression and CAGD can be found for example in [8–17].

On the other hand Gupta introduced a variant of classical Bernstein operator and similar modifications of well-known positive operators of Bernstein-type were studied by him, his collaborators and other researchers (see e.g. [18–25]).

It was an open problem to consider variants of Gupta-type for Shepard operators.

The aim of the present paper is to give a positive answer to the above question, introducing a generalization of Gupta-type of Shepard operator depending on a real positive parameter. Convergence results and uniform and pointwise approximation error estimates for such operator are given in Theorems 2.1–2.2 in Sect. 2.1. As a particular case, we obtain the first pointwise approximation error estimate for the original Shepard operator on equispaced mesh. Theorem 2.3 settles converse results and saturation statements for our operator. The corresponding proofs are based on direct estimates for the Shepard–Gupta-type operators.

In Sect. 2.2 an application to image compression is examined improving an analogous algorithm in [9] and numerical experiments confirming the outperformance of such technique compared with other algorithms are also shown.

## Results

For $n \in N$ consider the nodes matrix $X= ( x_{n,k}= x_{k}=k/n, k=0,\ldots ,n ) \subseteq [0,1]$. Then, for any function $f \in C([0,1])$ we denote by $S_{n}^{s}$ the Shepard operator defined by

1 $$ S_{n}^{s} ( X;f;x ) =S_{n}^{s}(f;x)= \frac{ \sum _{k=0}^{n} \frac{f( x_{k})}{\vert x-x_{k}\vert ^{s} } }{ \sum _{k=0}^{n} \frac{1}{\vert x-x_{k}\vert ^{s}} }, $$

with $x \in [0,1]$ and $s >0$ (cf. [26]). From (1) we deduce that $S_{n}^{s}$ is a positive, linear operator, preserving constants, interpolating *f* at $x_{k}$, $k=0,\ldots ,n$, and $S_{n}^{s}(f)$ is a rational function of degree $(sn,sn)$ for *s* even. Here we assume $s>2$ because of theoretical complications for $s\le 2$ (see, e.g., [3, 4]).

The approximation behavior of $S_{n}^{s}$ operator is well known and direct and converse results, saturation statements and simultaneous approximation estimates not possible by polynomials and corresponding to several nodes meshes distributions can be found for example in [1, 2, 4–7, 13, 27]. Applications to scattered data interpolation problems, CAGD and image compression were also examined (see e.g. [8–16]).

On the other hand Gupta introduced variants of Bernstein-type operators, studying the approximation properties of such operators (see e.g. [17–25]).

In the following subsection we extend such an approach to $S_{n}^{s}$ and study Shepard–Gupta-type operators.

### Approximation by Shepard–Gupta-type operators

For any $\alpha \ge 1$ and $s>2$ let

2 $$\begin{aligned} G_{n}^{\alpha ,s} ( X;f;x ) & = G_{n}^{\alpha ,s}(f;x) \\ & = \frac{ \sum _{k=0}^{n} f(x_{k}) [ ( \sum _{l=0}^{n}\frac{1}{\vert x-x_{l}\vert ^{s\alpha }} ) ^{\frac{1}{\alpha }}- ( \sum _{{l=0\atop l\ne k}}^{n}\frac{1}{\vert x-x_{l}\vert ^{s\alpha }} ) ^{\frac{1}{\alpha }} ] }{ \sum _{k=0}^{n} [ ( \sum _{l=0}^{n}\frac{1}{\vert x-x_{l}\vert ^{s\alpha }} ) ^{\frac{1}{\alpha }}- ( \sum _{{l=0\atop l\ne k}}^{n}\frac{1}{\vert x-x_{l}\vert ^{s\alpha }} ) ^{\frac{1}{\alpha }} ] }, \end{aligned}$$

with $x \in [0,1]$. From the definition it follows immediately that $G^{1,s}_{n}=S_{n}^{s}$, i.e. for $\alpha =1$, we find back the original Shepard operator (1). Moreover, $G_{n}^{\alpha ,s}$ is a positive, linear operator of interpolatory-type and is stable in the Fejér sense, i.e., $\forall x \in [0,1]$,

$$ \min_{0\le x\le 1} \bigl\vert f(x)\bigr\vert \le \bigl\vert G_{n}^{\alpha ,s}(f;x) \bigr\vert \le \max_{0\le x\le 1} \bigl\vert f(x)\bigr\vert . $$

We remark that Gupta variants of Bernstein-type operators depend on a positive parameter, not appearing in the kernel basis; here the parameter *α* appears both in the kernel basis $\vert x-x_{l} \vert ^{-s\alpha }$, both in the exponents in the inner summations at the r.h.s. in (2).

If we denote by $x_{j}$ the closest knot to *x*, with $x_{j} \le x \le x_{j+1}$, then $f(x_{j})$ (and also of $f(x_{ j+1 })$ if $x=(x_{j}+x_{j+1})/2$) influences $G_{n}^{\alpha ,s}(f;x)$ in a small neighborhood of *x* strongly—the “strong local control property”—as a consequence of the large value of $1/ {(x-x_{j})^{s\alpha }} $ in that range compared with the other terms. Consequently for *n* and *s* fixed and *α* increasing, $G_{n}^{\alpha ,s}(f;x)$ tends continuously to the step function

3 $$ \mathbb{S}(x)= \textstyle\begin{cases} f(x_{j}), & x_{j} \le x < x_{j+1/2}; \\ \frac{f(x_{j})+f(x_{j+1})}{2}, & x =x_{j+1/2}; \\ f(x_{j+1}), & x_{j+1/2} < x \le x_{j+1}, \end{cases} $$

with $x_{j+1/2}=(j+1/2)/n$. Analogously we can work for $x_{j}$ the closest knot to *x*, with $x_{j-1}\le x \le x_{j}$.

By such asymptotic behavior we can use the operator $G_{n}^{\alpha ,s}$ to successfully compress images expressed by piecewise constants (see Sect. 2.2).

Now we show that we can use $G_{n}^{\alpha ,s}$ to approximate functions from $C([0,1])$. Indeed, let $\Vert f \Vert $ be the usual supremum norm on $[0,1]$ of $f \in C([0,1])$ and $\omega (f)$ the usual modulus of continuity of *f*. Moreover, *C*, $C_{1}$ are positive constants possibly having different values even in the same formula; we say that $a \sim b$ iff $\vert a/b \vert \le C$ and $\vert b/a \vert \le C_{1}$.

#### Theorem 2.1

*Let* $\alpha \ge 1$. *Then*, *for any* $f \in C([0,1])$ *and* $n\in \mathbb{N}$,

4 $$ \bigl\Vert f-G_{n}^{\alpha ,s}(f) \bigr\Vert \le C \omega \biggl( f;\frac{1}{n} \biggr) . $$

#### Remark 2.1

Estimate (4) yields the uniform convergence, as $n\to \infty $, of $G_{n}^{\alpha ,s}(f)$ to $f, \forall f \in C([0,1]), \forall \alpha \ge 1$.

#### Proof

Since the $G_{n}^{\alpha ,s}$ operator interpolates at $x_{k}$, $k=0,\ldots ,n$, let $x\ne x_{k}$, $k=0,\ldots ,n$. Then assume $x_{j}$ to be the closest knot to *x*, with $x_{j} < x <x_{j+1}$ (the case when $x_{j+1} $ is the closest knot to *x* can be treated analogously). Therefore

$$ \vert x-x_{j}\vert \le \frac{1}{2n}. $$

We have

$$\begin{aligned} \bigl\vert f(x)-G_{n}^{\alpha ,s}(f;x) \bigr\vert & = \frac{\vert \sum _{k=0}^{n} [f(x)- f(x_{k})] [ ( \sum _{l=0}^{n}\frac{1}{\vert x-x_{l}\vert ^{s\alpha }} ) ^{\frac{1}{\alpha }}- ( \sum _{{l=0\atop l\ne k}}^{n}\frac{1}{\vert x-x_{l}\vert ^{s\alpha }} ) ^{\frac{1}{\alpha }} ] \vert }{ \sum _{k=0}^{n} [ ( \sum _{l=0}^{n}\frac{1}{\vert x-x_{l}\vert ^{s\alpha }} ) ^{\frac{1}{\alpha }}- ( \sum _{{l=0\atop l\ne k}}^{n}\frac{1}{\vert x-x_{l}\vert ^{s\alpha }} ) ^{\frac{1}{\alpha }} ] } \\ & \le \frac{ \omega ( f;\vert x-x_{j}\vert ) [ ( \sum _{l=0}^{n}\frac{1}{\vert x-x_{l}\vert ^{s\alpha }} ) ^{\frac{1}{\alpha }}- ( \sum _{{l=0\atop l \ne j}}^{n}\frac{1}{\vert x-x_{l}\vert ^{s\alpha }} ) ^{\frac{1}{\alpha }} ] }{ \sum _{k=0}^{n} [ ( \sum _{l=0}^{n}\frac{1}{\vert x-x_{l}\vert ^{s\alpha }} ) ^{\frac{1}{\alpha }}- ( \sum _{{l=0\atop l\ne k}}^{n}\frac{1}{\vert x-x_{l}\vert ^{s\alpha }} ) ^{\frac{1}{\alpha }} ] } \\ & \quad {}+ \frac{ \sum _{{k=0\atop k \ne j}}^{n} \omega ( f;\vert x-x_{k}\vert ) [ ( \sum _{l=0}^{n}\frac{1}{\vert x-x_{l}\vert ^{s\alpha }} ) ^{\frac{1}{\alpha }}- ( \sum _{{l=0\atop l\ne k}}^{n}\frac{1}{\vert x-x_{l}\vert ^{s\alpha }} ) ^{\frac{1}{\alpha }} ] }{ \sum _{k=0}^{n} [ ( \sum _{l=0}^{n}\frac{1}{\vert x-x_{l}\vert ^{s\alpha }} ) ^{\frac{1}{\alpha }}- ( \sum _{{l=0\atop l\ne k}}^{n}\frac{1}{\vert x-x_{l}\vert ^{s\alpha }} ) ^{\frac{1}{\alpha }} ] }. \end{aligned}$$

Since for $b< a, \eta \in (b,a)$ and $\alpha \ge 1$,

5 $$ a^{1/\alpha } - b^{1/\alpha } = \frac{a-b}{ \alpha \eta ^{1-1/\alpha } } \in \biggl( \frac{a-b}{ \alpha a^{1-1/\alpha } } , \frac{a-b}{ \alpha b ^{1-1/\alpha } } \biggr) , $$

working as usual (see e.g. [2]), it follows that

$$ \begin{aligned} \Biggl( \sum _{l=0}^{n} \frac{1}{\vert x-x_{l}\vert ^{s\alpha }} \Biggr) ^{\frac{1}{\alpha }}- \Biggl( \sum _{{l=0\atop l\ne k}}^{n}\frac{1}{\vert x-x_{l}\vert ^{s\alpha }} \Biggr) ^{\frac{1}{\alpha }}& \le \frac{\frac{1}{\vert x-x_{k}\vert ^{s\alpha }}}{ \alpha ( \sum _{{l=0\atop l \ne k}}^{n}\frac{1}{\vert x-x_{l}\vert ^{s\alpha }} ) ^{(\alpha -1)/\alpha }} \\ & \le \frac{C}{\alpha \vert x-x_{k}\vert ^{\alpha s}n^{s\alpha -s}}. \end{aligned} $$

Moreover,

$$ \frac{1}{\sum _{k=0}^{n} [ ( \sum _{l=0}^{n}\frac{1}{\vert x-x_{l}\vert ^{s\alpha }} ) ^{\frac{1}{\alpha }} - ( \sum _{{l=0\atop l\ne k}}^{n}\frac{1}{\vert x-x_{l}\vert ^{s\alpha }} ) ^{\frac{1}{\alpha }} ] } \le \frac{1}{ ( \sum _{l=0}^{n}\frac{1}{\vert x-x_{l}\vert ^{s\alpha }} ) ^{\frac{1}{\alpha }}- ( \sum _{{l=0\atop l\ne j}}^{n}\frac{1}{\vert x-x_{l}\vert ^{s\alpha }} ) ^{\frac{1}{\alpha }}}. $$

Again by (5)

6 $$\begin{aligned} \Biggl( \sum_{l=0}^{n}\frac{1}{\vert x-x_{l}\vert ^{s\alpha }} \Biggr) ^{\frac{1}{\alpha }}- \Biggl( \sum_{{l=0\atop l\ne j}}^{n}\frac{1}{\vert x-x_{l}\vert ^{s\alpha }} \Biggr) ^{\frac{1}{\alpha }} & \ge \frac{1/\vert x-x_{j}\vert ^{s\alpha }}{ \alpha ( \sum _{l=0}^{n}\frac{1}{\vert x-x_{l}\vert ^{s\alpha }} ) ^{(\alpha -1)/\alpha }} \\ & := \Sigma . \end{aligned}$$

Hence by (6)

$$\begin{aligned} \frac{1}{\Sigma}& = \alpha \vert x-x_{j}\vert ^{s\alpha } \Biggl( \sum _{l=0}^{n}\frac{1}{\vert x-x_{l}\vert ^{s\alpha }} \Biggr) ^{1-1/\alpha } \\ & \le \alpha \vert x-x_{j}\vert ^{s\alpha (1-1/\alpha +1/\alpha )} \Biggl( \frac{1}{\vert x-x_{j}\vert ^{s\alpha } }+ \sum _{{l=0\atop l \ne j}}^{n} \frac{1}{\vert x-x_{l}\vert ^{s\alpha }} \Biggr) ^{(\alpha -1)/\alpha } \\ & \le \alpha \vert x-x_{j}\vert ^{s} \Biggl( 1+\vert x-x_{j}\vert ^{s\alpha } \sum _{{l=0\atop l \ne j}}^{n} \frac{1}{\vert x-x_{l}\vert ^{s\alpha }} \Biggr) ^{(\alpha -1)/\alpha } \\ & \le C \alpha \vert x-x_{j}\vert ^{s}. \end{aligned}$$

Finally, collecting the above estimations, working as usual (see e.g. [2])

$$\begin{aligned} \bigl\vert f(x)-G_{n}^{\alpha ,s}(f;x)\bigr\vert &\le C \Biggl[ \omega \bigl( f; \vert x-x_{j} \vert \bigr) +\frac{\vert x-x_{j}\vert ^{s}}{n^{s\alpha -s}} \sum_{{k=0\atop k \ne j}}^{n} \frac{\omega ( f;\vert x-x_{k}\vert ) }{ \vert x-x_{k}\vert ^{\alpha s} } \Biggr] \\ &\le C \omega \biggl( f;\frac{1}{n} \biggr) . \end{aligned}$$

 □

Moreover, a pointwise approximation error estimate can be deduced.

#### Theorem 2.2

*Let* $\alpha \ge 1$. *Then*, *for any* $f \in C([0,1])$, $n\in \mathbb{N}$ *and for any* $x \in [0,1]$,

$$ \bigl\vert f(x)-G_{n}^{\alpha ,s}(f;x)\bigr\vert \le C \omega \bigl(f; \vert x-x_{j}\vert \bigr), $$

*with* $x_{j}$ *the closest knot to x*.

#### Remark 2.2

From Theorem 2.2, for $\alpha =1$, we obtain

7 $$ \bigl\vert f(x)-S_{n}^{s}(f;x)\bigr\vert \le C \omega \bigl(f; \vert x-x_{j}\vert \bigr). $$

This is the first pointwise estimate for Shepard operator on an equispaced mesh and it reflects the interpolatory character of $G_{n}^{\alpha ,s}$ at the knots $x_{k}$, $k=0,\ldots ,n$ and the constants preservation property. A similar estimate was obtained for a generalization of Shepard operator in [9]. The result in (7) is interesting; indeed the Shepard operator is strongly influenced by the mesh distribution and pointwise error estimates, for Shepard operators on nonuniformly spaced meshes present a function depending on the mesh thickness at the r.h.s. (see e.g. [2, 4]); to the contrary for the equispaced case pointwise estimates as in [2, 4] are against nature.

#### Proof

Following the proof of Theorem 2.1 we have

$$\begin{aligned} \bigl\vert f(x)-G_{n}^{\alpha ,s}(f;x)\bigr\vert & \le C \biggl\{ \omega \bigl(f;\vert x-x_{j} \vert \bigr)+ \frac{\vert x-x_{j}\vert ^{s}}{n^{\alpha s-s}} \frac{\omega (f;\vert x-x_{j+1}\vert )}{\vert x-x_{j+1}\vert ^{\alpha s}} \\ & \quad {} + \frac{\vert x-x_{j}\vert ^{s}}{n^{\alpha s-s}} \sum_{{x\ne j\atop j+1}} \frac{\omega (f;\vert x-x_{k}\vert )}{\vert x-x_{k}\vert ^{\alpha s}} \biggr\} . \end{aligned}$$

Obviously

$$\begin{aligned} \frac{\vert x-x_{j}\vert ^{s}}{n^{\alpha s-s}} \frac{\omega (f;\vert x-x_{j+1}\vert )}{\vert x-x_{j+1}\vert ^{\alpha s}} & \le C \frac{\vert x-x_{j}\vert ^{s}}{n^{\alpha s-s}}\frac{\omega (f;\vert x-x_{j}\vert )}{\vert x-x_{j}\vert \vert x-x_{j+1}\vert ^{\alpha s-1}} \le C\omega \bigl(f;\vert x-x_{j}\vert \bigr). \end{aligned}$$

Moreover, since $x-x_{k} > (j-k)/n$, $k=0,\ldots ,j-1$,

$$ \begin{aligned} \frac{\vert x-x_{j}\vert ^{s}}{n^{\alpha s-s}} \sum _{k=0}^{j-1} \frac{\omega (f;\vert x-x_{k}\vert )}{\vert x-x_{k}\vert ^{\alpha s }} & \le C\vert x-x_{j}\vert ^{s}\omega \biggl( f;\frac{1}{n} \biggr) \sum _{k=0}^{j-1} \frac{(j-k)n^{s}}{(j-k)^{\alpha s}} \\ & \le C\vert x-x_{j}\vert ^{s} n^{s} \omega \biggl( f;\frac{1}{n} \biggr) \\ & \le C\vert x-x_{j}\vert ^{s} n^{s} \omega \biggl( f;\frac{\vert x-x_{j}\vert }{n\vert x-x_{j}\vert } \biggr) \\ & \le C\vert x-x_{j}\vert ^{s} n^{s} \biggl( 1+\frac{1}{n\vert x-x_{j}\vert } \biggr) \omega \bigl( f;\vert x-x_{j}\vert \bigr) \\ & \le C\omega \bigl( f;\vert x-x_{j}\vert \bigr). \end{aligned} $$

Similarly we work for $k=j+1,\ldots ,n$.

Collecting all estimates, the assertion follows. □

Finally, we present the converse results for our operators.

#### Theorem 2.3

*If* $f \ne \mathrm{constant}$

8 $$ \limsup_{n \rightarrow \infty } \frac{\Vert G_{n}^{\alpha ,s}(f)-f \Vert }{\omega (f; 1/ {n})} \sim 1, $$

*where the sign* ∼ *does not depend on f*. *Moreover*

9 $$\begin{aligned}& \bigl\Vert G_{n}^{\alpha ,s}(f)-f \bigr\Vert =o\biggl( \frac{1}{n} \biggr)\quad \iff \quad f=\mathrm{constant}, \end{aligned}$$

10 $$\begin{aligned}& \bigl\Vert G_{n}^{\alpha ,s}(f)-f \bigr\Vert =O \biggl( \frac{1}{n} \biggr) \quad \iff \quad \omega (f;t) \le C t. \end{aligned}$$

#### Remark 2.3

First we observe that estimation (8) is a counterpart of (4) and is the analogous in some senses of the relation by Totik [28],

$$ \bigl\Vert B_{n}(f)-f \bigr\Vert \sim \omega ^{2}_{\psi } \biggl( f ; \frac{1}{\sqrt{n}} \biggr) , $$

with $B_{n}$ the classical Bernstein operator, $f \in C([0,1])$ and $\omega ^{2}_{\psi }$ the second order modulus of smoothness of Ditzian and Totik where $\psi (x)=\sqrt{x(1-x)}$. On the other hand, due to the interpolating behavior of $G_{n}^{\alpha ,s}$, we cannot have the estimation (8) with “lim” (instead of “lim sup”) because of a result stated in [3, p. 77] (cf. also [7, Theorem 2.1, p. 310]).

From (8) we deduce that direct estimate (4) cannot be improved.

Combining estimation (8) with the equivalence relation (see, e.g. [29]) $\omega (f;t) \sim K(f;t)$, with $K(f)$ the K-functional allows one to characterize such K-functionals.

Finally, the saturation problem for $G_{n}^{\alpha ,s}$ is settled by Eqs. (9)–(10).

#### Proof

We start to prove (8). From (2) we can write the operator $G_{n}^{\alpha ,s}$ as

$$\begin{aligned}& G_{n}^{\alpha ,s}(f;x) = \sum_{k=0}^{n} g_{k}(x)f(x_{k}), \\& g_{k}(x) = \frac{ [ ( \sum _{l=0}^{n}\frac{1}{\vert x-x_{l}\vert ^{s\alpha }} ) ^{\frac{1}{\alpha }}- ( \sum _{{l=0\atop l\ne k}}^{n}\frac{1}{\vert x-x_{l}\vert ^{s\alpha }} ) ^{\frac{1}{\alpha }} ] }{ \sum _{k=0}^{n} [ ( \sum _{l=0}^{n}\frac{1}{\vert x-x_{l}\vert ^{s\alpha }} ) ^{\frac{1}{\alpha }}- ( \sum _{{l=0\atop l\ne k}}^{n}\frac{1}{\vert x-x_{l}\vert ^{s\alpha }} ) ^{\frac{1}{\alpha }} ] }. \end{aligned}$$

Now if we verify that

11 $$\begin{aligned}& G_{n}^{\alpha ,s}(f;x)=f(x), \quad \mbox{if } f= \mathrm{constant}, \end{aligned}$$

12 $$\begin{aligned}& \sum _{\vert x-x_{k}\vert \ge d_{0}} \bigl\vert g_{k}(x)\bigr\vert =o \biggl( \frac{1}{n} \biggr) , \quad d_{0} >0 \mbox{ arbitrarily fixed}, \end{aligned}$$

13 $$\begin{aligned}& g_{j}(x) >1/2, \quad \mbox{if } \vert x-x_{j}\vert \le \frac{\delta }{n}, \quad 0 \le \delta < d_{1}< 1, \end{aligned}$$

14 $$\begin{aligned}& \sum _{k \ne j }\vert x-x_{k} \vert \bigl\vert g_{k}(x) \bigr\vert \le d_{2} \frac{\delta ^{1+\epsilon }}{n}, \quad \delta \mbox{ as above}, \end{aligned}$$

with $x_{j}$ again the closest knot to *x* and with certain positive fixed reals $d_{1}$, $d_{2}$, *ϵ*, then by using ([30, Theorem 2.1]) it follows that

15 $$\begin{aligned}& \limsup_{n \to \infty } n \bigl\Vert G_{n}^{\alpha ,s} (f)-f \bigr\Vert >CM(f), \\& M(f)= \sup_{x} \biggl( M(f;x);M(f;x):=\limsup _{\tau \to x} \frac{\vert f(\tau ) -f(x)\vert }{\vert \tau -x\vert } \biggr) . \end{aligned}$$

First we prove (11)–(14). We deduce Eq. (11) immediately by definition. Following the proofs of Theorems 2.1–2.2 we obtain

$$ \sum_{\vert x-x_{k}\vert >d_{0}} g_{k}(x) \le \frac{C}{n^{ \alpha s}} \sum_{\vert x-x_{k}\vert >d_{0}}\frac{1}{\vert x-x_{k}\vert ^{\alpha s}}\le \frac{C}{n^{\alpha s}} \frac{n+1}{d_{0}^{\alpha s}} =o \biggl( \frac{1}{n} \biggr) , $$

that is (12). Now we verify (13). Again working as in the proofs of Theorems 2.1–2.2,

$$\begin{aligned} \sum_{k \ne j } g_{k}(x) & \le \biggl[ \sum _{\vert x-x_{k} \vert \le 1}+\sum_{\vert x-x_{k} \vert \ge 1} \biggr] g_{k}(x) \\ & \le C \frac{\delta ^{s}n^{\alpha s}}{n^{\alpha s}} + C\frac{ \delta ^{s}n}{ n^{\alpha s} } \\ & \le C \delta ^{s} \biggl( 1+ \frac{1}{n^{\alpha s-1}} \biggr) \\ &\le \frac{1}{2} \end{aligned}$$

and by $g_{k} (x)\ge 0$ and $\sum g_{k}(x)=1$, (13) follows. Now we prove (14). Indeed

$$\begin{aligned} \sum _{k \ne j} \vert x-x_{k}\vert g_{k}(x) & \le C \frac{\vert x-x_{j}\vert ^{s}}{n^{\alpha s-s}} \biggl[ \sum _{ \vert x-x_{k}\vert \le 1 } +\sum _{\vert x-x_{k}\vert >1} \biggr] \frac{\vert x-x_{k}\vert }{\vert x-x_{k}\vert ^{\alpha s}} \\ & \le C\frac{\delta ^{s}}{n^{\alpha s}} \bigl[ n^{\alpha s-1} +n \bigr] \\ &\le C \frac{\delta ^{1+\epsilon }}{n}, \end{aligned}$$

i.e. we deduce (14). From (15) and (4) we have (cf. [7, p. 315])

16 $$\begin{aligned} C_{1}M(f) & \le n \bigl\Vert G_{n}^{\alpha ,s}(f) -f \bigr\Vert \le C_{2} n \omega \biggl( f; \frac{1}{n} \biggr) \\ & \le C_{2} \sup_{\tau \ne t}\frac{\vert f(\tau )-f(t)\vert }{\vert \tau -t\vert }:=C_{2}N(f). \end{aligned}$$

Now we recall that ([7, Lemma 3.1, p. 315])

$$ M(f)=N(f). $$

Therefore

17 $$ C_{1} M(f) \le C_{2} n \omega \biggl( f; \frac{1}{n} \biggr) \le C_{2} M (f) $$

and from (4), (16) and (17) we deduce (8). The proofs of (9) and (10) are omitted since they are analogous to the proof of Theorem 2.2 p. 316 in [7]. □

### Application to image compression

In this Section we apply the $G_{n}^{\alpha ,s}$ operator to a problem of image compression. An image can be considered from a mathematical point of view as a matrix of size $M\times N$ pixels, where the number of pixels affects resolution of an image and the size of he file that stores it (the higher the number of pixels, the better its resolution, the larger the file). As a degraded (compressed) image, we split the original image into consecutive blocks of size $\mathcal{B}\times \mathcal{B}$, choosing only the left-upper pixel from each block. We obtain a new image with a lower number of pixels ($M/\mathcal{B}\times N/\mathcal{B}$ pixels), and therefore a worse resolution and a smaller size of the file. The resulting compression ratio is $\rho \simeq \mathcal{B}^{2}$. We aim at decompressing the reduced image to rebuild the full resolution one. Since the sensors of the cameras are uniformly distributed according to a bidimensional grid, we need a bidimensional interpolation process based on equispaced mesh; in addition, for physical reasons related to the range of the color intensity of the red, green and blue components ($[0,1]$), it is preferable to rely on a positive operator. Therefore we consider the bidimensional operator $G_{M,N}^{\alpha ,s}(f)$ defined by

18 $$\begin{aligned} \begin{aligned} & G_{M,N}^{\alpha ,s}(f;x,y) = \sum _{k=1}^{M} \sum _{i=1}^{N}g_{k,M}(x) g_{i,N}(y)f(x_{k},y_{i}), \\ &g_{k,M}(x) = \frac{ ( \sum _{l=1}^{M}\frac{1}{\vert x-x_{l}\vert ^{s\alpha }} ) ^{\frac{1}{\alpha }}- ( \sum _{{l=1\atop l\ne k}}^{M}\frac{1}{\vert x-x_{l}\vert ^{s\alpha }} ) ^{\frac{1}{\alpha }} }{ \sum _{k=1}^{M} [ ( \sum _{l=1}^{M}\frac{1}{\vert x-x_{l}\vert ^{s\alpha }} ) ^{\frac{1}{\alpha }}- ( \sum _{{l=1\atop l\ne k}}^{M}\frac{1}{\vert x-x_{l}\vert ^{s\alpha }} ) ^{\frac{1}{\alpha }} ] } \\ &\hphantom{g_{k,M}(x) } = \frac{ ( \sum _{l=1}^{M} \prod _{j\ne l} \vert x-x_{j}\vert ^{s\alpha } ) ^{\frac{1}{\alpha }}- ( \sum _{{l=1\atop l\ne k}}^{M} \prod _{j\ne l} \vert x-x_{j}\vert ^{s\alpha } ) ^{\frac{1}{\alpha }} }{ \sum _{k=1}^{M} [ ( \sum _{l=1}^{M} \prod _{j\ne l} \vert x-x_{j}\vert ^{s\alpha } ) ^{\frac{1}{\alpha }}- ( \sum _{{l=1\atop l\ne k}}^{M} \prod _{j\ne l} \vert x-x_{j}\vert ^{s\alpha } ) ^{\frac{1}{\alpha }} ] }, \\ &g_{i,N}(y) = \frac{ ( \sum _{l=1}^{N}\frac{1}{\vert y-y_{l}\vert ^{s\alpha }} ) ^{\frac{1}{\alpha }}- ( \sum _{{l=1\atop l\ne i}}^{N}\frac{1}{\vert x-x_{l}\vert ^{s\alpha }} ) ^{\frac{1}{\alpha }} }{ \sum_{k=1}^{N} [ ( \sum_{l=1}^{N}\frac{1}{\vert y-y_{l}\vert ^{s\alpha }} ) ^{\frac{1}{\alpha }}- ( \sum_{{l=1\atop l\ne i}}^{N}\frac{1}{\vert y-y_{l}\vert ^{s\alpha }} ) ^{\frac{1}{\alpha }} ] } \\ &\hphantom{g_{k,M}(x) } = \frac{ ( \sum_{l=1}^{N}\prod_{j\ne l} \vert y-y_{j}\vert ^{s\alpha } ) ^{\frac{1}{\alpha }}- ( \sum_{{l=1\atop l\ne i}}^{N}\prod_{j\ne l} \vert y-y_{j}\vert ^{s\alpha } ) ^{\frac{1}{\alpha }} }{ \sum_{k=1}^{N} [ ( \sum_{l=1}^{N}\prod_{j\ne l} \vert y-y_{j}\vert ^{s\alpha } ) ^{\frac{1}{\alpha }}- ( \sum_{{l=1\atop l\ne i}}^{N}\prod_{j\ne l} \vert y-y_{j}\vert ^{s\alpha } ) ^{\frac{1}{\alpha }} ] }, \end{aligned} \end{aligned}$$

with $x,y\in [0,1]$, $x_{i}=(i-1)/(M-1)$, $i=1,\ldots ,M$, $y_{j}=(j-1)/(N-1)$, $j=1,\ldots ,N$. We observe that for computer calculations the nonbarycentric-type representations at the right hand side in (18) are suitable. We can write Eq. (18) as

$$ G_{M,N}^{\alpha ,s}(f;x,y) = \sum_{k=1}^{M} \Biggl[ \sum_{i=1}^{N} f(x_{k},y_{i})g_{i,N}(y) \Biggr] g_{k,M}(x). $$

This allows one to develop a two-step procedure, each one involving the same unidmensional operator of the type (2) applied first to the rows of the matrix of pixels and then to the columns of the matrix resulting after application of the first step (or vice versa).

We will compare the results obtained by the $G_{M,N}^{\alpha ,s}$ operator with bi-linear, bi-cubic and bi-spline methods. For the comparison we used the Signal-to-Noise Ratio, SNR, defined as

$$ \mathrm{SNR}=10 \log _{10} \frac{(2^{B}-1)^{2}}{\mathrm{MSE}}, $$

with *B* denoting the number of bits necessary to represent the intensity of the pixels and

$$ \mathrm{MSE}=\frac{1}{MN} \sum_{i=1}^{M} \sum_{j=1}^{N} (f_{ij} - \hat{f}_{ij})^{2}, $$

where $f_{ij}$ is the original image in the pixels *i*, *j*, $i=1,\ldots ,M$, $j=1,\ldots ,N$, and $\hat{f}_{ij}$ is the resulting image after decompression by the original bidimensional Shepard operator, $G_{M,N}^{\alpha ,s}$ operator, bi-linear, bi-cubic and bi-spline functions. The SNR compares the level of the compression error to the level of the signal: the higher SNR, the better the approximation of the original image.

By construction of the $G_{M,N}^{\alpha ,s}$ operators (cf. (3)) there are better approximate images that can be represented by piecewise constant functions; therefore a synthetic image having such a feature will be considered. We notice that tuning of the parameter *α* permits one to get a better approximation error.

**Figure 1:**
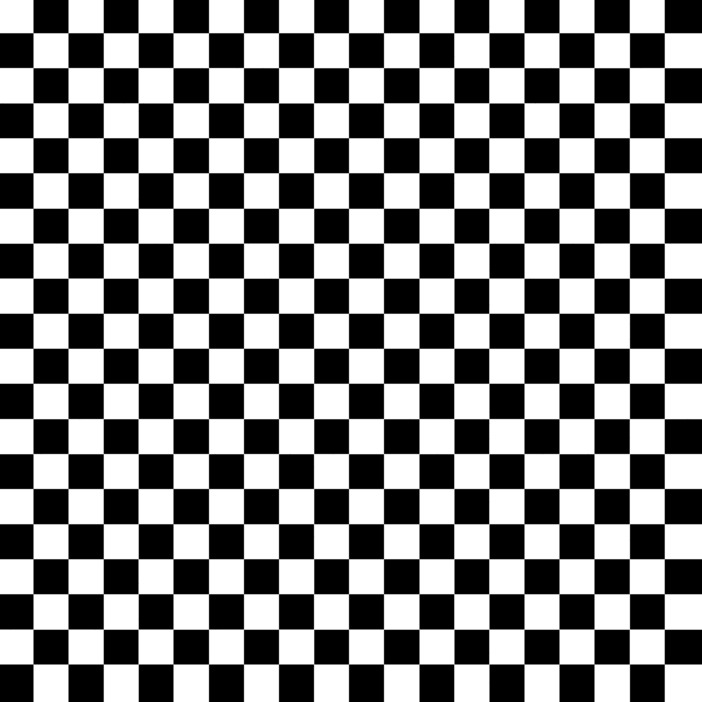
Image of chessboard chosen as a test example

According to the comment above we consider as an example of image a chessboard (Fig. 1) with 2048 pixels for both coordinates ($M=N=2048$) having 20 alternating boxes for each row or column of the chessboard. The usual 8-bit gray scale representation is considered for the color, so that $B=8$. We generated reduced resolution images at compression ratios $\rho =4,9,16,25,36$ ($\mathcal{B}=2,\ldots ,6$).

The value of SNR for bi-linear, bi-cubic, bi-spline, Shepard ($s=4,6$), $G_{M,N}^{\alpha ,4}$, $G_{M,N}^{\alpha ,6}$ operators, with $\alpha =1.1,1.3,2,3,5,10$ and compression ratio $\rho =4,9,16,25,36$, is shown in Table 1.

**Table 1:** SNR of the decompressed images for the Chessboard test example at compression ratio $\rho =4,9,16,25,36$ and for bi-linear, bi-cubic, bi-spline, Shepard ($s=4,6$), $G_{M,N}^{\alpha ,4}$, $G_{M,N}^{\alpha ,6}$ operators, $\alpha =1.1,1.3,2,3,5,10$. The higher SNR, the more accurate the methodology

Method	*ρ* = 4	*ρ* = 9	*ρ* = 16	*ρ* = 25	*ρ* = 36
Original Shepard (*s* = 4)	79.6	77.4	76.0	75.0	74.0
$G_{M,N}^{1.1,4}$	80.3	78.1	76.7	75.6	74.6
$G_{M,N}^{1.3,4}$	81.6	79.2	77.8	76.7	75.7
$G_{M,N}^{2,4}$	85.3	82.4	80.7	79.5	78.3
$G_{M,N}^{3,4}$	89.8	85.8	83.7	82.3	81.0
$G_{M,N}^{5,4}$	98.5	91.7	88.5	86.5	84.7
$G_{M,N}^{10,4}$	120.4	106.4	99.7	95.5	92.2
Original Shepard (*s* = 6)	82.0	79.6	78.1	77.0	76.0
$G_{M,N}^{1.1,6}$	82.9	80.3	78.8	77.7	76.6
$G_{M,N}^{1.3,6}$	84.4	81.6	80.0	78.8	77.7
$G_{M,N}^{2,6}$	89.1	85.3	83.3	82.0	80.6
$G_{M,N}^{3,6}$	95.6	89.8	87.0	85.2	83.6
$G_{M,N}^{5,6}$	108.8	98.5	93.7	90.7	88.3
Bi-linear	73.3	72.0	70.3	69.4	68.5
Bi-cubic	73.8	72.0	70.8	69.9	69.0
Bi-spline	73.2	71.4	70.2	69.3	68.4

We can see that the Shepard–Gupta-type operator (18) gives the best results at any compression ratio and that accuracy improves when *α* increases.

**Figure 2:**
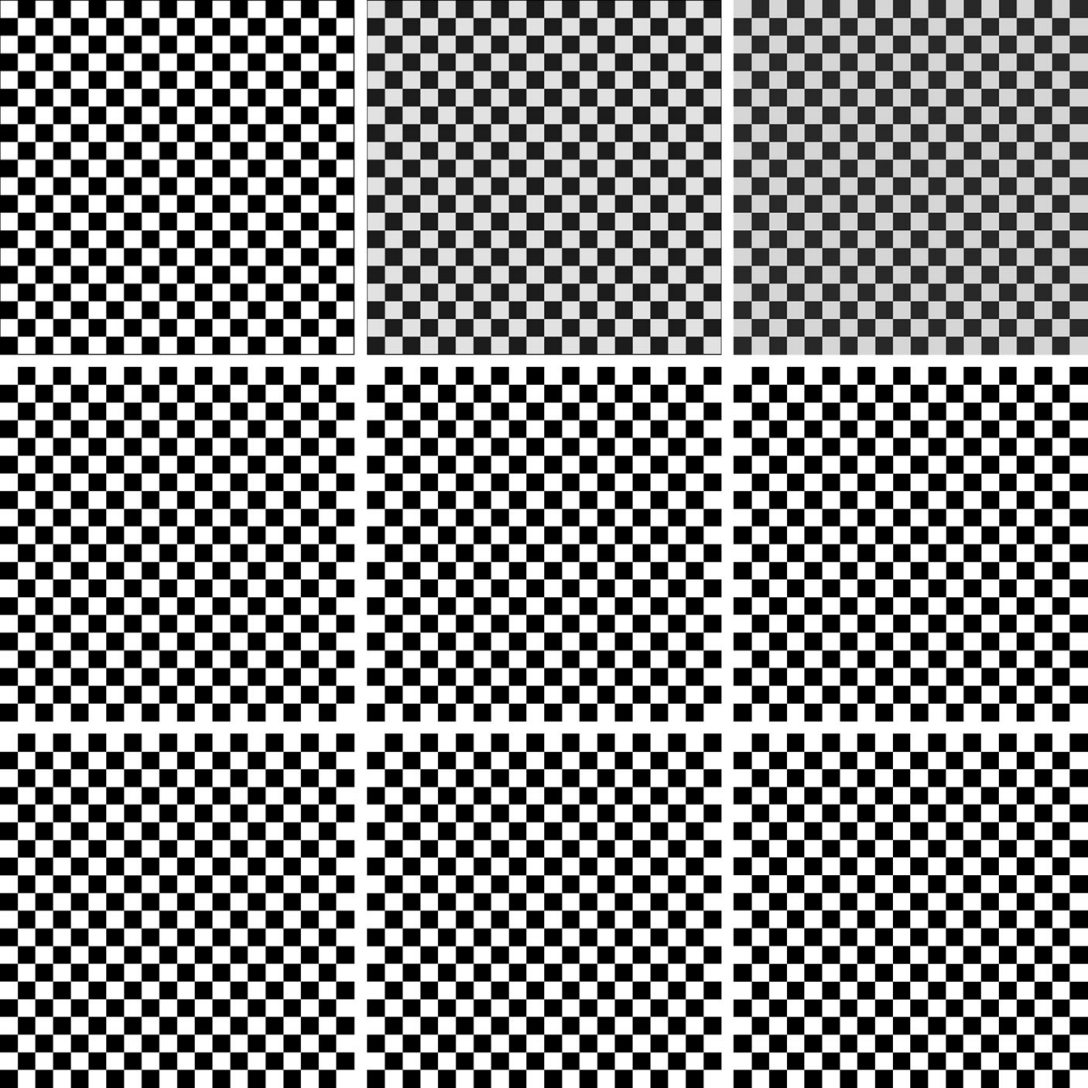
From top to bottom and left to right: the chessboard image decompressed by bi-linear, bi-cubic, bi-spline, Shepard ($s=4$), $G_{M,N}^{2,4}$, $G_{M,N}^{10,4}$, Shepard ($s=6$), $G_{M,N}^{2,6}$ and $G_{M,N}^{10,6}$ operators starting from the image compressed with ratio 25

Figure 2 shows the decompressed images for bi-linear, bi-cubic, bi-spline, Shepard ($s=4,6$), $G_{M,N}^{\alpha ,4}$, $G_{M,N}^{\alpha ,6}$ operators, $\alpha =2,10$, obtained for compression ratio 25. We notice the gray color of the truly white boxes in the chessboard for bi-spline and bi-cubic operators (middle and right upper plots). It is due to overshoots (pixels having intensities greater than 1) and undershoots (pixels with intensity less than 0). As is well known these artifacts are particularly deleterious for images. Bi-linear and Shepard–Gupta-type operators being stable in the Fejér sense do not suffer from this artifact.

**Figure 3:**
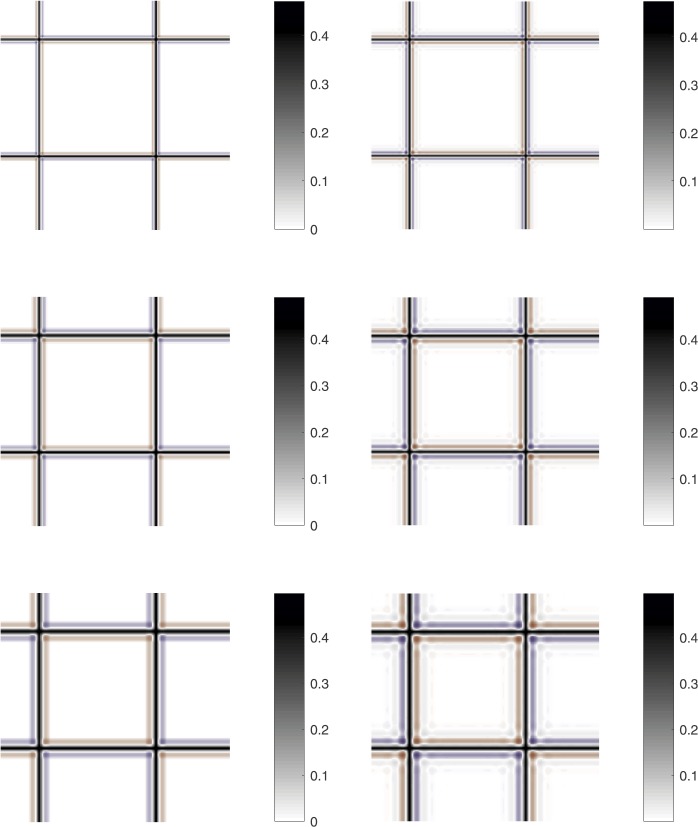
Error of the decompressed images for the chessboard test example for the considered methods (particular). From top to bottom and left to right bi-cubic and bi-spline for $\rho =9$, bi-cubic and bi-spline for $\rho =25$, bi-cubic and bi-spline for $\rho =49$. Blue and red colors indicate undershoots and overshoots, respectively

To better appreciate this artifact and differences among the above methodologies, Fig. 3 shows the (absolute) error of the decompressed images for only bi-cubic and bi-spline operators at different compression ratios ($\rho =9$, 25, 49), since the other operators are not affected by the overshoot-undershoot artifact. Overshoots and undershoots are represented with red and blue color, respectively.

**Figure 4:**
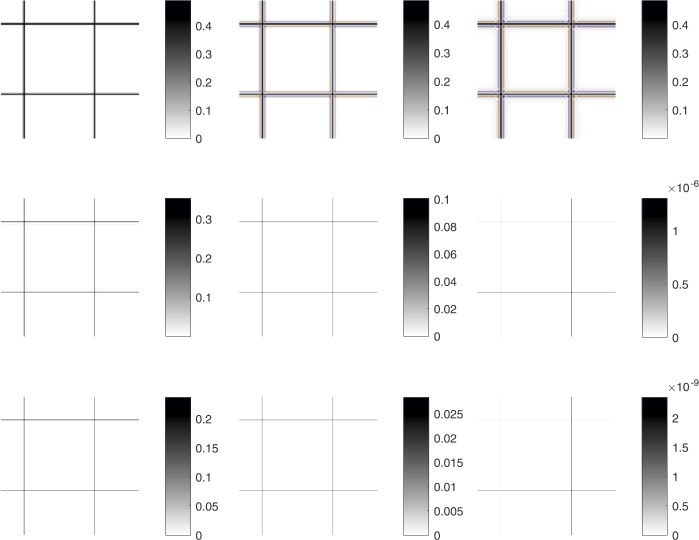
Error of the decompressed images for the Chessboard test example for the considered methods (particular). From top to bottom and left to right: bi-linear, bi-cubic, bi-spline, Shepard ($s=4$), $G_{M,N}^{2,4}$, $G_{M,N}^{10,4}$, Shepard ($s=6$), $G_{M,N}^{2,6}$, $G_{M,N}^{10,6}$ operators for compression ratio $\rho =25$. Blue and red colors indicate undershoots and overshoots, respectively

A full assessment of all considered methods is graphically given in Fig. 4 in a particularization of Fig. 3. The figure shows the smaller error (higher SNR) achieved by the Shepard–Gupta-type method.

## Conclusions

The paper gives a positive answer to the problem to extend the Bézier variant technique introduced and studied by Gupta for the well-known linear positive operators of Bernstein-type, to the Shepard interpolator operator, widely used in rational approximation and scattered data interpolation problems. The authors construct and study the Shepard–Gupta-type operator and settle convergence results, uniform and pointwise approximation error estimates, converse theorems and saturation statements, improving in some sense analogous results for the original Shepard-type operator. The peculiar asymptotic behavior of the Shepard–Gupta-type operator allows one to successfully compress images represented by piecewise constants, improving previous algorithms.
